# Polymer coordination promotes selective CO_2_ reduction by cobalt phthalocyanine†
†Electronic supplementary information (ESI) available: Representative cyclic voltammograms of modified electrodes, representative current–time plots from controlled potential electrolyses, and tabulated results from control experiments. See DOI: 10.1039/c5sc04015a


**DOI:** 10.1039/c5sc04015a

**Published:** 2016-02-02

**Authors:** W. W. Kramer, C. C. L. McCrory

**Affiliations:** a Division of Chemistry and Chemical Engineering , California Institute of Technology , Pasadena , CA 91125 , USA . Email: cmccrory@umich.edu

## Abstract

Cobalt phthalocyanine (**CoPc**) is a known electrocatalyst for the carbon dioxide reduction reaction (CO_2_RR) that, when adsorbed onto edge-plane graphite (EPG) electrodes, shows modest activity and selectivity for CO production along with co-generation of H_2_. In contrast, electrodes modified with **CoPc** immobilized in a poly-4-vinylpridine (P4VP) film show dramatically enhanced activity and selectivity compared to those modified with **CoPc** alone. **CoPc-P4VP** films display a faradaic efficiency of ∼90% for CO, with a turnover frequency of 4.8 s^–1^ at just –0.75 V *vs.* RHE. Two properties of P4VP contribute to enhancing the activity of **CoPc**: (1) the ability of individual pyridine residues to coordinate to **CoPc** and (2) the high concentration of uncoordinated pyridine residues throughout the film which may enhance the catalytic activity of **CoPc** through secondary and other outer coordination sphere effects. Electrodes modified with polymer-free, five-coordinate **CoPc(py)** films (py = pyridine) and with **CoPc** catalysts immobilized in non-coordinating poly-2-vinylpyridine films were prepared to independently investigate the role that each property plays in enhancing CO_2_RR performance of **CoPc-P4VP**. These studies show that a synergistic relationship between the primary and outer coordination sphere effects is responsible for the enhanced catalytic activity of **CoPc** when embedded in the P4VP membrane.

## Abstract 2

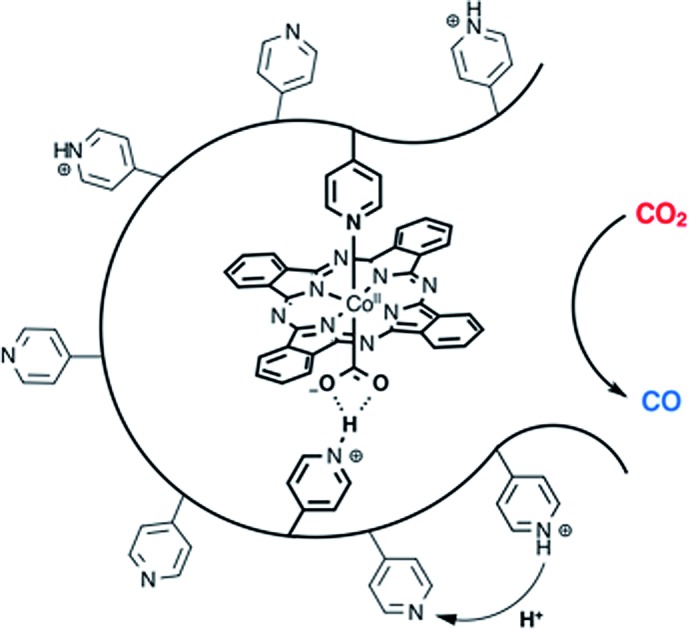
We present a study of the mechanistic factors that transform cobalt phthalocyanine from a catalyst that shows modest performance for the electrocatalytic reduction of CO_2_ to CO production into one of the most active and selective molecular catalyst reported in aqueous media when immobilized in a poly-4-vinylpyridine membrane

## Introduction

The selective electrochemical reduction of CO_2_ into fuels is an important strategy for the development renewable energy sources. Polycrystalline Cu is currently the state-of-the-art heterogeneous electrocatalyst for carbon dioxide reduction reactions (CO_2_RR), producing 2–6 e^–^ products at potentials as positive as ∼–0.9 V *vs.* RHE in aqueous neutral, and near-neutral solutions.^1^–^6^ However, Cu-catalyzed CO_2_RR tends to yield a large distribution of carbon containing products, and the operating potentials are quite negative compared to the thermodynamic potentials of the major products.

In contrast, molecular catalysts show propensity to form a single, typically 2–4 e^–^ CO_2_ reduction products.^7^–^23^ Cobalt phthalocyanine (**CoPc**) is a well-known molecular CO_2_RR catalyst.^24^–^32^ When adsorbed to graphite electrodes, **CoPc** is reported to reduce CO_2_ to CO in aqueous solution. However there is significant co-generation of H_2_ produced from the reduction of protons in the aqueous electrolyte. Previous studies have shown that incorporating **CoPc** into a poly-4-vinylpyridine (P4VP) membrane increases the catalyst’s selectivity for the production CO from CO_2_ over the evolution of H_2_ from water.^33^,^34^ The exact mechanisms for the observed P4VP-enhanced activity and selectivity for CO_2_ reduction remain unclear. Two properties of the P4VP membrane are believed to contribute to the enhancement of **CoPc** as a CO_2_RR catalyst. First, individual pyridine residues of P4VP can coordinate to the square planar cobalt center of **CoPc**. Axial coordination of pyridine has been implicated in enhanced CO_2_RR activity for **CoPc** and cobalt porphyrin catalysts.^25^,^26^,^28^,^33^,^34^ Second, the uncoordinated pyridine residues throughout the membrane, which form the secondary and outer coordination spheres of **CoPc**, also may contribute to the increased activity. In acidic solution some proportion of the pyridine residues will be protonated,^33^,^35^,^36^ and the presence of these protonated residues may enable secondary coordination sphere effects such as hydrogen bonding interactions that can stabilize activated intermediates, and outer sphere effects like the availability of protons around catalyst active sites. However, the mechanism by which P4VP enhances CO_2_RR by **CoPc** has not yet been explicitly studied. It is important to determine how P4VP increases the activity and selectivity of **CoPc** for CO_2_ reduction, as such information could aid designs of improved immobilized molecular CO_2_RR catalyst systems that may find wider applications in the emerging field of solar-fuels technology.

In the current work, the properties of P4VP are assessed independently in order determine how each affects CO_2_RR by **CoPc**. Electrodes modified with **CoPc** and **CoPc-P4VP** were studied using controlled potential electrolysis (CPE) to better understand the base activity of **CoPc** and the increase in activity that coincides with P4VP encapsulation. As previously reported, large increases in activity and selectivity were observed for **CoPc-P4VP** over the parent **CoPc**. We present more a detailed evaluation of CPE data to provide a more comprehensive study of their properties. In particular, we find that **CoPc-P4VP** is remarkably active for the selective generation of CO, operating in pH 5 solution with an average current density of 2 mA cm^–2^ and a turnover frequency of 4.8 s^–1^ with nearly 90% faradaic efficiency for CO production at –1.25 V *vs.* SCE (–0.73 V *vs.* RHE). This operating potential is only –0.61 V from the thermodynamic potential of CO production (*E*^0^ = –0.125 V *vs.* RHE) making the **CoPc-P4VP** system among the most active molecular catalyst reported for the selective reduction of CO_2_ to any single product in aqueous solution.^34^,^37^–^44^

**Fig. 1:**
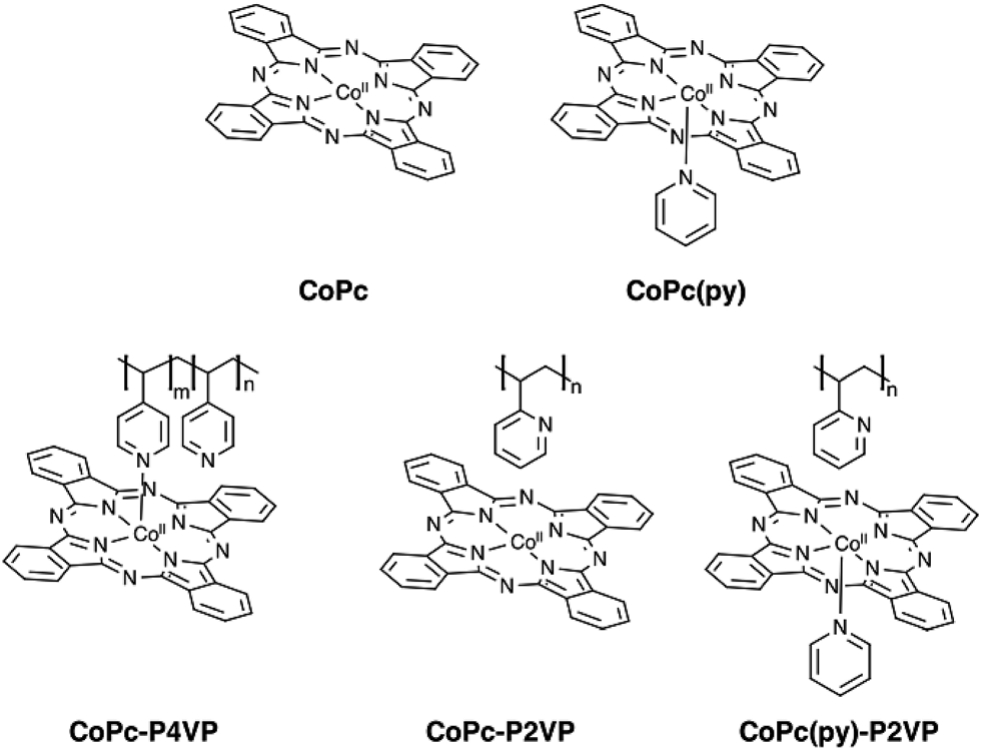
Proposed molecular structures of **CoPc**, **CoPc-P4VP**, **CoPc(py)**, **CoPc-P2VP**, and **CoPc(py)-P2VP**.

To investigate the influence of axial coordination of **CoPc** on the CO_2_RR activity in the absence of the polymer film, electrodes modified with axially coordinated **CoPc(py)** were prepared from deposition solutions of **CoPc** in the presence of pyridine. Likewise, to separate the intrinsic properties of the polymer from axial coordination effects, electrodes with four-coordinate **CoPc**, and five-coordinate **CoPc(py)** encapsulated in a non-coordinating poly-2-vinylpyridine (P2VP) membrane were prepared (Fig. 1). Our results suggest that there is a synergistic relationship between axial coordination and the chemical environment imposed by the P4VP membrane that leads to dramatic enhancements in activity observed for **CoPc-P4VP**.

## Results

### **CoPc** and **CoPc-P4VP**

Electrodes modified with **CoPc** and **CoPc-P4VP** were prepared to investigate the CO_2_RR catalytic activity of the free catalyst and the effect of the P4VP support. Films were deposited onto an EPG disc electrode by casting a solution in DMF on the electrode surface and allowing the solvent to evaporate at room temperature. Cyclic voltammograms (CVs) of both films show a broad Co(ii/i) reduction between –0.4 and –0.7 V *vs.* SCE, and a more well defined phthalocyanine centered reduction at *ca.* –0.9 V *vs.* SCE (Fig. S1 and S2†). Because the peaks in the CVs were quite broad, the electrochemically active coverage of the catalyst could not be readily determined. Therefore, the total deposited coverage of 1.3 × 10^–9^ mol cm^–2^, calculated from the 5 μL of 0.05 mM **CoPc** solution deposited on each 5 mm diameter electrode, was used in all subsequent calculations. This loading of **CoPc** was held constant for each deposited film in this study.

**Fig. 2:**
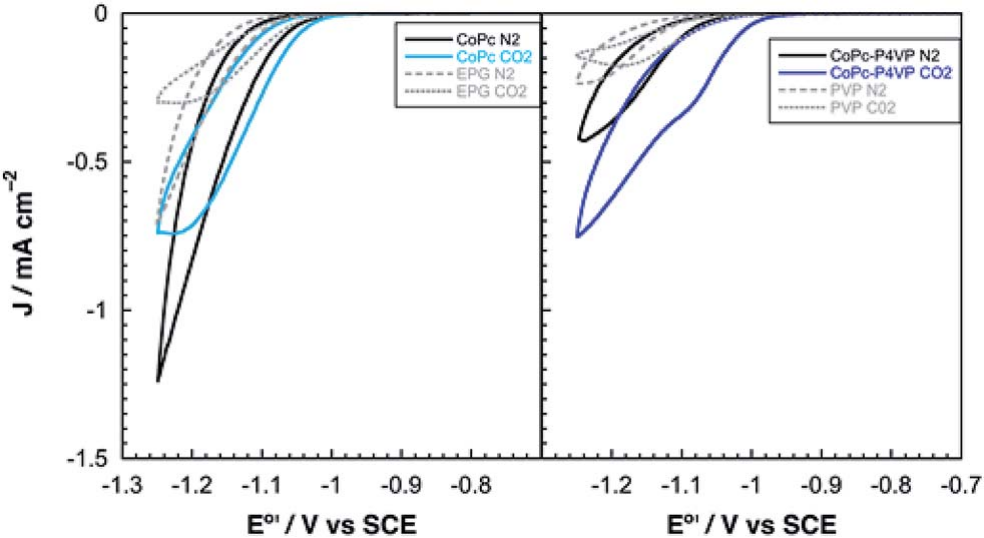
Representative static cyclic voltammograms of (left) **CoPc** and (right) **CoPc-P4VP** under N_2_ and CO_2_.

**Fig. 3:**
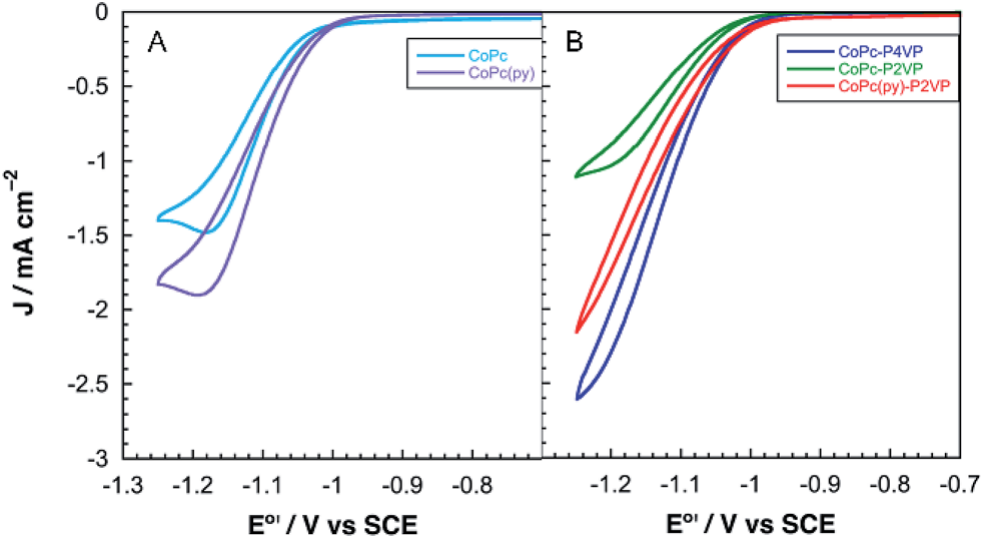
Representative rotating disk electrode voltammograms at 1600 rpm of (A) the free catalysts **CoPc** (light blue) and **CoPc(py)** (purple) and (B) polymer immobilized catalysts **CoPc-P4VP** (dark blue), **CoPc-P2VP** (green) and **CoPc(py)-P2VP** (red) under an atmosphere of CO_2_.

Cobalt phthalocyanine is known to be a competent HER catalyst.^45^–^48^ Under an atmosphere of N_2_, scans negative of the phthalocyanine reduction show the onset of a catalytic wave attributed to HER at approximately –1.04 V *vs.* SCE for **CoPc** modified electrodes and –1.08 *vs.* SCE for electrodes modified with **CoPc-P4VP**. Under a CO_2_ atmosphere, the onset of the catalytic wave shifts slightly positive to –1.0 V *vs.* SCE, for both films as shown in Fig. 2. The peak current in the presence of CO_2_ of both films in static CVs is approximately 0.75 mA cm^–2^. When performed at 1600 rpm (to ensure steady state delivery of substrate to the electrode surface), rotating disk electrode voltammograms (RDEVs) of **CoPc-P4VP** display much greater peak currents than **CoPc** (Fig. 3A and B, respectively). The more negative onset potential for H_2_ evolution with **CoPc-P4VP** modified electrodes suggests that the P4VP film suppresses HER compared to free **CoPc**. However, the onset of the catalytic wave under CO_2_ was not affected by the presence of the polymer film indicating that P4VP does not similarly suppress CO_2_RR activity of **CoPc**.

**Fig. 4:**
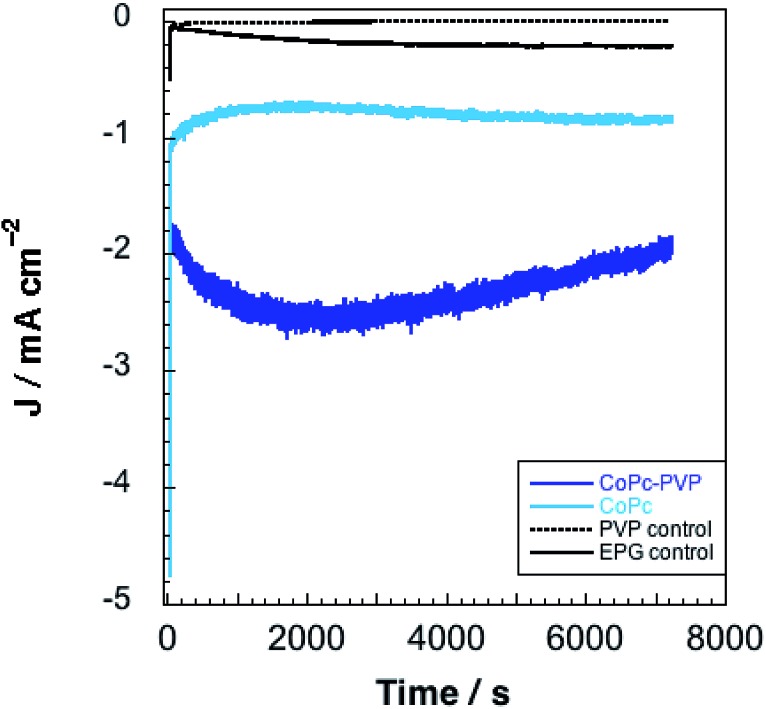
Representative electrolyses for **CoPc**, **CoPc-P4VP**, EPG, and EPG-P4VP conducted at –1.25 V *vs.* SCE.

CPE experiments were performed at –1.25 V *vs.* SCE to assess faradaic efficiencies for CO_2_ reduction by **CoPc** and **CoPc-P4VP** modified electrodes (Fig. 4). Electrolyses were conducted for 2 h in a gas-tight, two-compartment electrolysis cell under a CO_2_ atmosphere in a constantly stirred, CO_2_ saturated, 0.1 M aqueous NaH_2_PO_4_ buffer solution at pH 4.7. The results of these experiments are summarized in Table 1. Nearly four times as much charge was passed using **CoPc-P4VP** modified electrodes compared to **CoPc** modified electrodes. Over the 2 h experiments, current densities averaging 0.62 mA cm^–2^ and 2.0 mA cm^–2^ were observed for **CoPc** and **CoPc-P4VP**, respectively. The only products observed for both films were CO and H_2_. No liquid products were detected for either **CoPc** or **CoPc-P4VP** within our detection limits (∼10 μM). Faradaic efficiencies (*ε*) for CO are 36% for **CoPc** and 89% for **CoPc-P4VP**. *ε*_H_2__ is 41% for **CoPc** and only 5% for **CoPc-P4VP** films. Turnover numbers (TON) for CO of 4500 and 34 000 were determined for **CoPc** and **CoPc-P4VP**, respectively. From these TONs, empirical turnover frequencies (TOFs) could be determined for the production of CO (Table 1). It is important to note that, because the total deposited catalyst coverage was used to determine TON and TOF values, these values are likely a lower limit of the actual values. While the previous study of **CoPc** immobilized in 100% P4VP did not report faradaic efficiency data for **CoPc** and **CoPc-P4VP** films, selectivity data is reported in the form of the ratio of CO to H_2_.^33^ For **CoPc**, the observed CO/H_2_ ratio of 0.9 : 1 is in good agreement with the previously reported ratio of ∼1.5 : 1. However, in our hands, H_2_ production by **CoPc-P4VP** is significantly reduced. The previous study reported a CO/H_2_ ratio of 4 : 1, while we observe a CO/H_2_ ratio of 19 : 1. This difference in selectivity could be due to a difference in P4VP membrane thickness—5 μL of the 1% w/v P4VP deposition solution to prepare the modified electrodes in this study whereas only 2 μL was used in the earlier report.

**Table 1:** Results obtained from 2 h CPE experiments at –1.25 V *vs.* SCE for **CoPc** modified electrodes. The reported values are averages of measurements from at least three experiments with independently prepared electrodes. Errors are given as standard deviations except for those for *ε*_total_ which were calculated as standard errors

	Charge/C	*ε*_CO_	TON_CO_ (2 h)	TOF_CO_ (s^–1^)	*ε*_H_2__	*ε*_total_
**CoPc**	0.58 ± 0.24	36 ± 7%	4.5 ± 2.4 × 10^3^	0.6 ± 0.3	41 ± 8%	77 ± 10%
**CoPc(py)**	0.83 ± 0.48	68 ± 3%	1.2 ± 0.7 × 10^4^	1.6 ± 1.0	19 ± 5%	87 ± 6%
**CoPc-P4VP**	1.9 ± 0.20	89 ± 3%	3.4 ± 0.4 × 10^4^	4.8 ± 0.6	5 ± 1%	94 ± 3%
**CoPc-P2VP**	0.36 ± 0.08	73 ± 8%	5.6 ± 1.8 × 10^3^	0.8 ± 0.2	12 ± 3%	85 ± 9%
**CoPc(py)-P2VP**	1.76 ± 0.27	83 ± 5%	3.0 ± 0.5 × 10^4^	4.2 ± 0.7	6 ± 5%	89 ± 7%

A series of control experiments were conducted to ensure that the observed CO production was due to the electrocatalytic reduction of CO_2_ by **CoPc**. The results of these control experiments are provided in Tables S1–S3.† To summarize, potentiostatic electrolysis experiments were conducted with unmodified and P4VP coated EPG electrodes. For EPG electrodes under 1 atm CO_2_, the major product was hydrogen. For P4VP coated EPG electrodes under 1 atm CO_2_, hydrogen was also observed, but in much smaller amounts compared with the bare EPG electrodes due to the very low charge passed during the experiments. In both cases only very small amounts of CO could be observed (0.17 μmol), less than 2% of the amount of CO generated by **CoPc-P4VP** under identical conditions. Additionally, electrolyses were conducted with **CoPc** and **CoPc-P4VP** under an atmosphere of N_2_. As expected the only product observed was H_2_ for both systems.

### Axial ligand effect

To investigate the influence of axial coordination on CO_2_RR activity of **CoPc** in the absence of the polymer film, electrodes modified with the pyridine coordinated catalyst, **CoPc(py)**, were prepared using a deposition solution of **CoPc** in neat pyridine. A large excess of the axial ligand was employed in the deposition solutions to ensure the equilibrium would favor the axially coordinated species.^49^ RDEVs of the **CoPc(py)** films under an atmosphere of CO_2_, at 1600 rpm, display a positive shift in the onset potential of the catalytic wave, compared to **CoPc**, and a significant increase in the peak catalytic current over the parent complex, as shown in Fig. 3A. During 2 h electrolysis experiments, **CoPc(py)** films passed ∼1.5 times more charge than **CoPc**, with average current of 0.96 mA cm^–2^ (Fig. S6†). Faradaic efficiencies for CO with **CoPc(py)** were more than double the parent catalyst at 68%. Only 19% of the total charge passed went towards the production of H_2_ in the **CoPc(py)** film. No liquid products were detected. The TON for CO with the five-coordinate complex was around 12 000, with a TOF_CO_ of 1.6 s^–1^.

Enhanced CO_2_RR performance has been observed with some heterogeneous catalysts after the addition of free pyridine to the electrolysis solution.^50^–^52^ CPE experiments were conducted with **CoPc** films in the presence of added pyridine and 2,6-lutidine to determine whether the presence of free ligand increases CO_2_RR activity. The results of these experiments are summarized in the ESI (Table S3†). Electrolyses preformed using **CoPc** modified electrodes with 0.05 mM added pyridine or 2,6-lutidine showed no significant change in the amount of charge passed or in the faradaic efficiencies for CO or H_2_ compared to **CoPc** alone. The concentration of added pyridine and 2,6-lutidine in these experiments is orders of magnitude higher than the concentration of pyridine that could be achieved from the complete dissociation of pyridine from **CoPc(py)** into solution (6.3 nM). Because no increase in activity or selectivity was observed with the addition of free pyridine, it appears that the axial ligand remains bound to **CoPc** in films of **CoPc(py)** during catalysis.

### Polymer encapsulation

To separate the secondary and outer coordination sphere effects of the polymer membrane from the axial coordination effects, modified EPG electrodes were prepared with **CoPc** encapsulated in a poly-2-vinylpyridine (P2VP) membrane. Steric congestion around the pyridine nitrogens in P2VP should prevent coordination to **CoPc**.^33^ Though P2VP is not capable of coordinating, it should maintain the secondary and outer coordination sphere properties of P4VP. **CoPc-P2VP** films were deposited under the same conditions as described for the deposition of **CoPc-P4VP**.

RDEVs of **CoPc-P2VP** under CO_2_ show the onset of catalysis at potentials similar to **CoPc**, with peak currents lower than those of the parent catalyst (Fig. 3B). Measurements of CO_2_ catalysis showed that **CoPc-P2VP** films passed much less charge during the electrolyses than **CoPc-P4VP**, and the average current density was 0.39 mA cm^–2^. However, **CoPc-P2VP** operated with *ε*_CO_ = 73%, much higher than **CoPc**, and *ε*_H_2__ for **CoPc-P2VP** was only 12%. Compared to **CoPc**, **CoPc-P2VP** showed practically identical TON_CO_ and TOF_CO_ during the 2 h electrolyses. These experiments indicate that while the P2VP support does not increase the activity of **CoPc** for CO_2_ reduction, it does suppress HER.

Electrodes modified with **CoPc(py)** dispersed in a P2VP membrane (**CoPc(py)-P2VP**) were prepared to reintroduce the axial ligand to **CoPc** in the P2VP catalysts films. These films were deposited from a 19 : 1 DMF/pyridine solution containing 0.05 mM **CoPc** and 1% w/v P2VP. Incorporating a more active, five-coordinate **CoPc** catalyst in the P2VP film increased CO_2_RR activity by nearly an order of magnitude. The onset potential of CO_2_RR catalysis in RDEVs of **CoPc(py)-P2VP** occurred at nearly the same potential as **CoPc-P4VP**, and the peak current was much larger than **CoPc-P2VP** (Fig. 3B). Average current densities of 1.9 mA cm^–2^ were observed for the **CoPc(py)-P2VP** system, which are nearly the same as for **CoPc** encapsulated in P4VP. The total charge passed in bulk electrolyses increased to 1.7 C for **CoPc(py)-P2VP** from 0.36 C with **CoPc-P2VP**. Faradaic efficiency for CO was increased from 73% without pyridine to 83%, and *ε*_H_2__ decreased to 6%. By reintroducing an axial ligand to **CoPc** in P2VP the CO_2_RR performance was restored to the level observed for **CoPc-P4VP**.

## Discussion

**Fig. 5:**
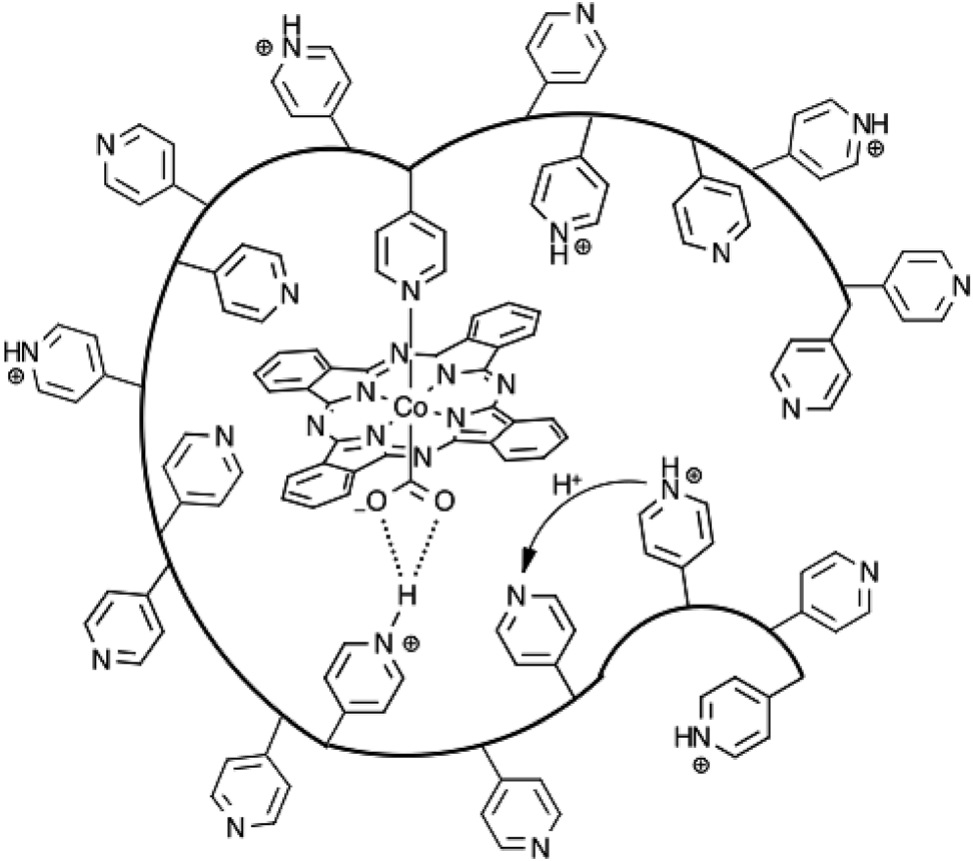
An illustration of **CoPc** immobilized in P4VP. Three properties of the polymer membrane which are suggested to be important to the activity and selectivity of **CoPc-PVP** are illustrated. Pyridine residues that can coordinate to **CoPc**, the ability for uncoordinated pyridine residues to act as proton relays, and the hydrogen bonding interactions that may occur between protonated pyridines and activated CO_2_.

In agreement with previous reports, the encapsulation of **CoPc** in P4VP results in a dramatic increase of activity and selectivity for CO_2_RR over free **CoPc**. The present study examines the activities and product distributions of these catalyst films in greater detail in an effort to better understand the underlying cause of this phenomenon. By itself, **CoPc** is an unremarkable CO_2_RR catalyst that displays poor selectivity and relatively low activity for the reduction of CO_2_ over proton reduction. Once immobilized in P4VP, the selectivity for CO production jumps to nearly 90% with close to an order of magnitude increase in the TON_CO_. Under the conditions described herein, **CoPc-P4VP** displays an even higher selectivity for CO production over HER than has been previously reported.^33^ In fact, **CoPc-P4VP** is among the most active and selective molecular CO_2_ reduction catalysts yet studied in aqueous solutions (Table 2). Additionally, at –0.73 V *vs.* RHE, or a 0.61 V overpotential for the production of CO, the high activity and selectivity are notable, even in comparison to heterogeneous catalysts like copper, which at this potential non-selectively produces CO and formate with lower faradaic efficiencies along with a large amount of H_2_ under similar conditions,^1^–^6^ and gold, which is among the most active and selective polycrystalline metal catalysts for the reduction of CO_2_ to CO.^5^,^53^,^54^ It is clear that the P4VP membrane alters the chemical environment of **CoPc** in a way that promotes the reduction of CO_2_. We hypothesize that both the ability of P4VP to coordinate **CoPc**, and the secondary and outer coordination sphere effects that arise from the partial protonation of free pyridine residues throughout the polymer film are the major contributors to the increased activity, as illustrated in Fig. 5. Though polymer encapsulation is a common way of immobilizing molecular catalysts onto an electrode surface,^19^,^41^,^55^–^64^ the use of a polymer membrane that offers this combination of properties is rare.^25^,^39^,^48^,^56^,^65^–^69^

**Table 2:** A comparison of reported molecular CO_2_RR electrocatalysts which display high activity and/or selectivity in aqueous media to **CoPc-P4VP** and **CoPc(py)-P2VP**

Catalyst	Activity/mA cm^–2^	V *vs.* RHE	pH	Products (*ε*)	TOF/s^–1^	Ref.
**CoPc-P4VP**	2.0 ± 0.2	–0.73	4.7	CO (89 ± 3%), H_2_ (5 ± 1%)	CO: 4.8	This study
**CoPc(py)-P2VP**	1.9 ± 0.2	–0.73	4.7	CO (83 ± 5%), H_2_ (6 ± 5%)	CO: 4.2	This study
**CoPc**-(90% P4VP, 10% polystyrene)/BPG	NR	–0.70	4.4	CO (71.6%), H_2_ (21.0%)	CO: 3.1^*a*^ (EA 41)^*b*^	34
**CoPc**-(90% P4VP, 10% polystyrene)/BPG	NR	–0.66	6.8	CO (77.2%), H_2_ (16.6%)	CO: 2.9^*a*^ (EA 51)^*b*^	34
COF-367-Co	∼3.5	–0.67	7.3	CO (91%), H_2_ (20%)	CO: 0.05^*a*^ (EA 0.5)^*b*^	37
COF-367-Co(1%)	∼0.5	–0.67	7.3	CO (48%), H_2_ (51%)	CO: 0.2^*a*^ (EA 2.6)^*b*^	37
[Mn(bpy(*t*Bu)_2_)(CO)_3_Br]/Nafion/MWCNT	0.2	–0.75	7	CO (46%), H_2_ (44%)	CO: 0.0005	38
Ni(cyclam)-PALA^*c*^	NA	–0.17	8	CO (92%)	NA	39
Poly(Cr(vinylterpy)_2_)	NR^*d*^	–0.52	5.8^*e*^	HCHO (87%)	NR^*d*^	40
Re[(bpy)(CO_3_)Br]/Nafion	0.002	–0.65	7	HCO_2_H (48%), CO (16.5%), H_2_ (39%)	CO: 0.002, HCO_2_H: 0.006	41
Co(Ch)/MWCNT	NR	–0.83	4.6	CO (89%)	CO: 0.04	42
Ir-Pincer (2^MeCN^)	0.60	–1.0	6.95	HCOOH (93%), H_2_ (7%)	NR^*f*^	43
Ni(cyclam)	0.64–0.97	–0.67	5	CO (84 ± 4%)	NA	44
Ni(MTC)	0.64–0.97	–0.67	5	CO (88 ± 7%)	NA	44
Ni(MCC)	0.64–0.97	–0.67	5	CO (92 ± 2%)	NA	44
Ni(HTC)	0.64–0.97	–0.67	5	CO (88 ± 7%)	NA	44

^*a*^These TOF values were recalculated from the literature report using the total loading of catalyst cast onto the surface, as opposed to the amount detected by CV. We believe using the total amount of catalyst deposited provides a more accurate comparison to other reported values in the literature.

^*b*^Reported TOF values based on the electroactive surface coverage of the catalyst.

^*c*^Solution phase catalyst at 2.0 mg mL^–1^ concentration.

^*d*^No time information was provided for the electrolysis in the report, so activity and TOF could not be calculated for the electrolysis. However, the reported Koutecky–Levich analysis in the manuscript yields a TOF of 5.2 s^–1^.

^*e*^Estimated pH of 0.1 M NaClO_4_ saturated with CO_2_.

^*f*^No empirical TOF based on electrolysis data was provided. However, the authors did report a TOF value calculated from CV data of 7.3 s^¬1^.

**Fig. 6:**
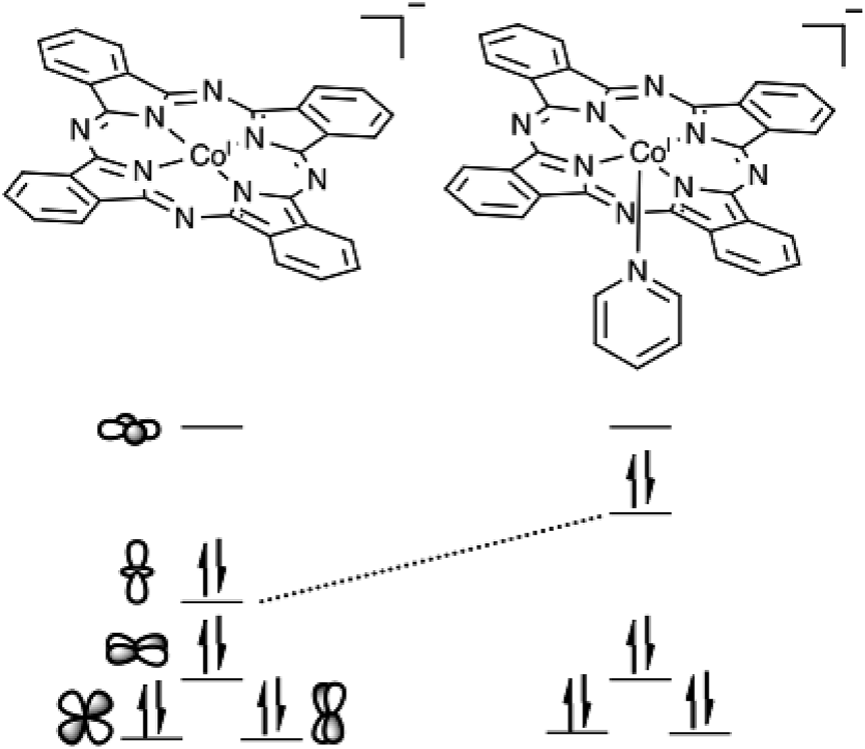
Relative energies of the cobalt d orbitals in the 1 e^–^ reduced forms of **CoPc** and **CoPc(py)**. Shown is the energy increase of the cobalt d_z^2^_ orbital that results the coordination of the axial pyridine.

Porphyrin and phthalocyanine catalysts often display enhanced catalytic activity upon the coordination of an axial ligand.^26^,^28^,^70^–^72^ The ability of P4VP to act as an axial ligand contributes to the enhanced CO_2_RR activity and selectivity of **CoPc-P4VP** modified electrodes. Other than in a P4VP film, the only previous study that suggests an axial ligand effect on CO_2_RR selectivity with **CoPc** comes from an IR spectroelectrochemical study which suggests that **CoPc** films deposited from neat pyridine are more selective for CO_2_RR over HER than films deposited from THF.^73^ The enhanced activity of **CoPc(py)** shows that the coordinating ligand effect is responsible for the increases in the rate of CO_2_ reduction. As shown in Fig. 6, axial coordination raises the energy of the cobalt d_z^2^_ orbital. When the metal center is reduced to Co(i), filling the d_z^2^_ orbital, the metal becomes a stronger nucleophile, and is better able to bind and activate the Lewis acidic carbon of CO_2_. The significant decrease in *ε*_H_2__ with **CoPc(py)** compared to **CoPc** may be attributable to the CO_2_ binding outcompeting proton reduction. This also suggests that CO_2_ reduction takes place *via* a CO_2_ binding event rather than the formation of a cobalt hydride that then goes on to react with CO_2_,^26^,^34^ as the formation of such a hydride would presumably also be the first step in HER.

The primary and outer coordination spheres of enzyme active sites are specially adapted to efficiently perform difficult catalytic transformations for the production of a single desired product. Protein film voltammetry has been used to demonstrate that enzymes adsorbed on electrode surfaces can perform electrocatalytic reactions, including CO_2_RR, with very high TON,^74^,^75^ although low surface coverages limit the overall activity. These studies have also established the importance of electron and proton relays in these reactions.^76^–^78^ In molecular systems, the importance of the hydrogen bonding in the secondary coordination sphere to stabilize reactive intermediates has been demonstrated by several groups.^79^–^85^ In particular, second sphere proton relays incorporated into a ligand scaffold can lead to high electrocatalytic activity in reactions that require the delivery of multiple protons.^86^–^89^ The P4VP membrane imbues many of these secondary coordination sphere effects to **CoPc** without the need for synthetically challenging ligand modifications.

While **CoPc-P2VP** showed virtually no increase in TON for CO over free **CoPc**, HER activity was greatly diminished. Combined with the decrease in HER performance displayed by **CoPc-P4VP** under N_2_ and in the EPG-P4VP control experiments, it is clear that the polymer film helps to suppress proton reduction pathways. It may be that limited diffusion of water through the film is the reason for the lower activity for HER. In the case of polymer free **CoPc** modified electrodes, the catalyst is directly exposed to solution where there is a large local concentration of protons. In the polymer immobilized systems, the catalyst is not exposed to the solvent directly and the permeability of water through the PVP membrane at this pH is limited, so the main source of available protons in the polymer film is likely from protonated pyridine residues.^33^,^35^,^36^ At pH 4.7 approximately 20% of the pyridine residues in the film are protonated.^33^ It is reasonable to conclude that increase in selectivity for CO over H_2_ with **CoPc-P2VP** compared to **CoPc** alone could be due to a weak acid effect from the protonated pyridine residues.^90^ While these pyridinium residues may be acidic enough to act as a proton donor to activated CO_2_ intermediates, they may also be basic enough to suppress HER activity. The selectivity for CO with **CoPc-P4VP** is much higher than with **CoPc-P2VP**. This suggests that in addition to the outer sphere weak acid effect, the primary sphere effect of axial coordination on the selectivity for CO_2_RR over HER, demonstrated by **CoPc(py)** is also an important factor for this catalytic selectivity.

**Fig. 7:**
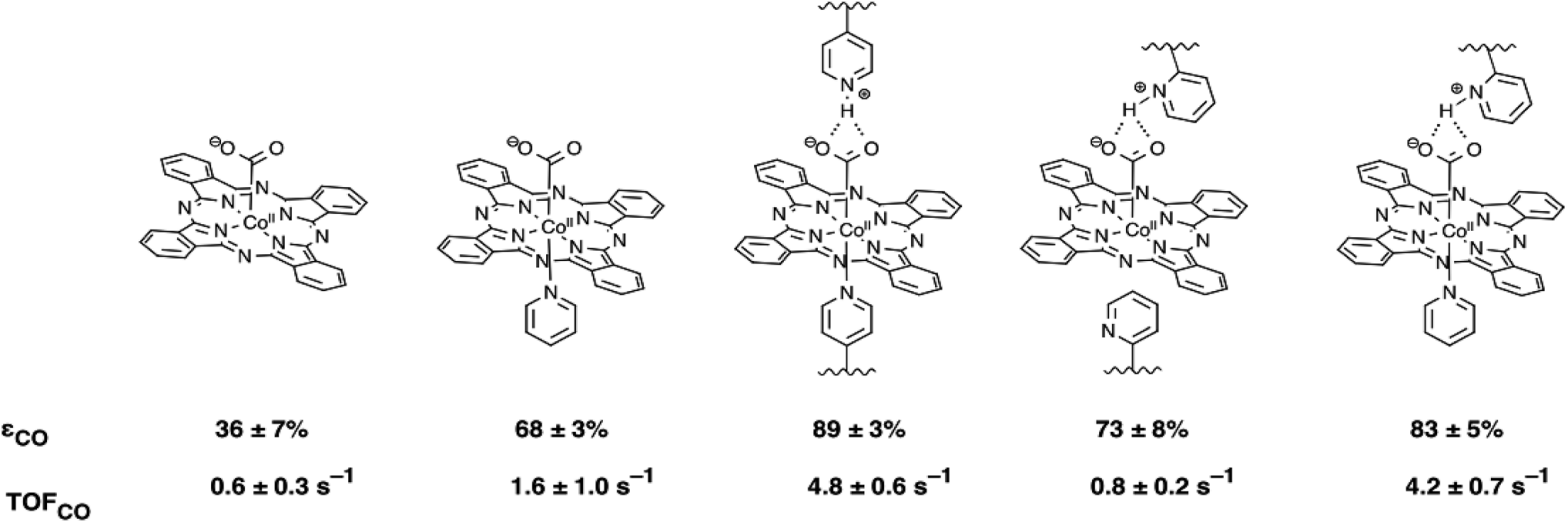
Proposed activated CO_2_ complexes and secondary coordination sphere interaction for each catalyst film studied. Also shown are the faradaic efficiencies and turnover frequencies for each catalyst system determined from CPE measurements.

Beyond limiting HER activity, the polymer membrane effects additional CO_2_RR rate enhancements, but only for the more active five-coordinate **CoPc** catalysts **CoPc-P4VP** and **CoPc(py)-P2VP**. Protonated pyridine residues in close proximity to the **CoPc**(L) catalyst active site (L = py, P4VP) may be able to stabilize the [(L)Co(ii)Pc-CO_2_]^–^ intermediate through hydrogen bonding interactions, and may increase the rate of proton transfer to the activated [(L)Co(ii)Pc-CO_2_]^–^ complex (Fig. 7). Again though, these secondary coordination sphere effects only result in rate enhancements with the five-coordinate **CoPc**(L) catalysts. This is demonstrated by comparing the observed TOF_CO_ of **CoPc-P2VP** and **CoPc(py)-P2VP** to the TOF_CO_ of the catalysts without the polymer film. No enhancement of CO_2_RR activity was observed when the four-coordinate **CoPc** was immobilized in the P2VP membrane compared to the catalyst outside of the polymer membrane. Alternatively, a large increase in TOF_CO_ was observed for **CoPc(py)-P2VP** compared to polymer free films of **CoPc(py)**. The chemical environment around the catalyst active sites in **CoPc-P2VP** and **CoPc(py)-P2VP** films is likely unaffected by the presence of the axial pyridine, yet only in the case of **CoPc(py)-P2VP** does being in this chemical environment lead to increased catalytic activity. A plausible explanation for this phenomenon is that the presence of the axial ligand may change the rate limiting step of CO_2_ reduction from the formation of Co(ii)Pc-CO_2_^–^ with the four-coordinate catalyst, to the subsequent proton transfer steps in the case of the five-coordinate catalyst. If this is the case, the secondary coordination sphere effects would have no influence of the rate of CO_2_ reduction by **CoPc**, but would result in the dramatic increases in CO_2_RR activity observed for the more active **CoPc**(L) catalysts.

## Conclusions

Immobilization of cobalt phthalocyanine in poly-4-vinylpyridine dramatically improves its activity as a catalyst for the reduction of CO_2_ to CO. The polymer membrane slows the competing HER catalytic pathway while also increasing rate of CO_2_RR compared to the polymer free catalyst. It is clear from the results obtained for **CoPc(py)** and **CoPc-P2VP** that neither the primary, or secondary and outer coordination sphere effects alone are responsible for the large increases in CO_2_RR activity. These effects must be combined, as is the case in **CoPc-P4VP** and **CoPc(py)-P2VP**, to produce a **CoPc** CO_2_RR catalyst that is highly active and selective. In this way **CoPc-P4VP** and **CoPc(py)-P2VP** mimic enzymes, where all aspects of the catalyst chemical environment are critical to the function of the system overall.

We propose that this electrode-modification strategy using functional polymer membranes can be applied more generally to the field of CO_2_ reduction catalysis. The ability of PVP films to suppress HER without inhibiting CO_2_RR is in itself of importance considering that the cogeneration of H_2_ by CO_2_ reduction catalysts, both molecular and heterogeneous, in aqueous media limits CO_2_RR product selectivity. Additionally, the incorporation of groups other than pyridine in the polymer chain could allow us to engineer the chemical environment around catalyst active sites in a facile way, without the need for complicated ligand synthesis. Each component of these catalyst films could be selected based on its ability to maximize the efficiency and selectivity of the system as a whole. This approach could lead to the development of new highly active catalyst films which are not only selective for a single highly reduced product, but also cost effective and easily prepared.

## Experimental

### Materials

Ultrapure water (18.2 MΩ cm resistivity) was purified with a Thermo Scientific Barnstead Nanopure water purification system. Carbon dioxide (CO_2_, Alphagaz–1 grade, 99.99%) and helium (He, Alphagaz–1 grade, 99.999%) were purchased from Air–Liquide and used as received. Nitrogen (N_2_) was boil-off gas from a liquid nitrogen source and used without further purification. **CoPc** (97%) was obtained from Alfa Aesar and used as received. P4VP (average *M*_w_ ∼ 160 000) and P2VP (average *M*_w_ ∼ 159 000) were purchased from Sigma Aldrich and used as received. *N*,*N*-Dimethylformamide (DMF, ACS grade) was purchased from VWR. Pyridine (ACS grade) was purchased from Alfa Aesar. Deuterium oxide (D_2_O, 99.9%) was purchased from Cambridge Isotope Labs, Inc.

### Preparation of modified electrodes

All catalyst films were prepared from deposition solutions containing 0.05 mM **CoPc**. The parent **CoPc** was deposited from solutions in DMF, as were the polymer encapsulated **CoPc** films. Deposition solutions for polymer-encapsulated **CoPc** films contained 1% w/v of the polymer. For films of **CoPc(py)**, neat pyridine was used as the solvent in place of DMF. Modified electrodes were prepared by evaporation of 5 μL of the deposition solution on an edge-plane graphite disc electrode (5 mm diameter, effective electrode area: 0.13 cm^2^, Pine Research Instrumentation). For all modified electrodes used in this study, the coverage of deposited **CoPc** was 1.3 × 10^–9^ mol cm^–2^. Prior to catalyst deposition, the electrodes were cleaned by manually polishing the surface with 600 grit Carbimet SiC grinding paper (Buehler) followed by sonication in ultrapure water for 10 min.

### Electrochemical methods

Experiments were conducted using a Bio-Logic SP200 potentiostat/galvanostat with a built-in electrochemical impedance spectroscopy (EIS) analyzer or a Bio-Logic VMP3 multichannel potentiostat/galvanostat with a built-in EIS analyzer. The modified working electrodes were mounted in a Pine Instrument Company E6-series ChangeDisk rotating disk electrode assembly in an MSR rotator. The auxiliary electrodes were carbon rods (99.999%, Strem), and the reference electrodes were commercial saturated calomel electrodes (SCE) (CH-Instruments) that were externally referenced to a solution of ferrocene monocarboxylic acid (Sigma-Aldrich) in a 0.2 M phosphate buffer at pH 7 (0.284 V *vs.* SCE).^91^ The supporting electrolyte solution was 0.1 M aqueous NaH_2_PO_4_ (BioUltra ≥ 99.0%, Sigma) with the pH adjusted to pH 5 by the addition of 1 M aqueous NaOH. When saturated with CO_2_ the pH of the electrolyte solution was measured to be 4.71. The pH of electrolyte solutions were measured with a VWR Symphony multiparameter meter with a Thermo Scientific Orion refillable Ag/AgCl pH electrode filled with Orion Ag/AgCl reference electrode filling solution. The pH meter was calibrated with a 4 point calibration curve at pH = 1.68, 4.00, 7.00, and 10.00. Data were recorded using the Bio-Logic EC-Lab software package.

### Electrochemical measurements

Cyclic voltammetry (CV) and rotating disc electrode (RDE) experiments were conducted in a glass two-chamber U-cell. One chamber held the working and reference electrodes in ∼150 mL of solution, and the other chamber held the auxiliary electrode in ∼20 mL of solution. The two chambers were separated by a fine-porosity glass frit. Both chambers were purged with either N_2_ or CO_2_ for ∼20 min prior to each experiment and then blanketed with N_2_ or CO_2_ during data collection. CO_2_ and N_2_ were water-saturated by bubbling though gas-washing bottle filled with ultrapure water.

### Controlled potential electrolyses

Experiments were conducted in a custom, two-chamber U-cell. The working and reference electrodes were held in a gas tight chamber with a total volume of either 81 or 91 mL and filled with solution to provide 41 mL of headspace. The second chamber held the auxiliary electrode in 15 mL of solution and was open to air. The two chambers were separated by a Nafion 117 cation exchange membrane (Aldrich). The chemically-modified working electrode was held in a Pine Instrument Company RDE internal hardware kit and mounted in a custom PEEK sleeve. Prior to each electrolysis experiment the cell was purged with CO_2_ for at least 1 h and then sealed under an atmosphere of CO_2_. All experiments were conducted at least three time with independently prepared electrodes. All values reported are the averages of these repetitions and errors are reported as standard deviations.

To allay the concern that the use of a carbon-rod auxiliary electrode with a Nafion cation exchange membrane might lead to the formation of formate that can cross over into the catholyte solution, and to validate the choice of experimental conditions, electrolyses were performed using platinum mesh electrode as the auxiliary and a Selemion AMV anion exchange membrane (AGC Engineering Co., Ltd). No difference in activity or product distribution was observed between electrolysis experiments using platinum mesh electrodes and Selemion ion exchange membranes compared to those using carbon rod electrodes and Nafion ion exchange membranes.

### Product detection and quantification

Gaseous products (*i.e.* CO and H_2_) in the headspace were quantified using an Agilent Technologies 7890A Permanent Gas and Hydrogen Analyzer GC system with two analyzer channels. Using a valve system, column configuration, and method developed by Agilent Technologies, gases were separated so that H_2_ was detected on one channel using an N_2_ carrier gas, and all other gases were detected on the second channel using a He carrier gas. All gases were detected with a thermal conductivity detector (TCD), and chromatographs were analyzed using the Agilent OpenLAB CDS ChemStation software. A Pressure-Lok A–2 gas-tight analytical syringe (10 mL, Valco VICI Precision Sampling, Inc.) was used to collect 8 mL aliquots from the working electrode chamber headspace. Prior to each injection, the sample loop was purged with N_2_, then an aliquot was injected directly into the 6 mL sample loop. The presence of liquid products in the catholyte was investigated using Presat solvent suppression NMR spectroscopy on a 400 MHz Bruker cryoprobe spectrometer using 900 μL of the catholyte solution containing 100 μL D_2_O for signal locking, and 3.2 mM DMF as an internal standard.^3^ Faradaic efficiencies were determined by dividing the total moles products for each product by the moles of electrons calculated from the amount of charge passed during the controlled-potential electrolysis measurements, accounting for the number of electrons required to produce each product.

## Supplementary Material

Supplementary information
