# Supplementary material for: Polymer coordination promotes selective CO2 reduction by cobalt phthalocyanine
Source: Chem Sci. 2016 Feb 2;7(4):2506–15. doi: 10.1039/c5sc04015a (PMC5477023; doi:10.1039/c5sc04015a)
Supplement: Supplementary file 1 [file SC-007-C5SC04015A-s001.pdf]

Electronic Supplementary Material (ESI) for Chemical Science.
This journal is © The Royal Society of Chemistry 2016

Polymer coordination promotes selective CO_2 reduction by cobalt phthalocyanine

Wesley W. Kramer[a], Charles C. L. McCrory[a,†,*]

[a] *California Institute of Technology, Pasadena, California*

[†]*Present Address: University of Michigan, Ann Arbor, Michigan*

-- Supporting Information--

Table of Contents

Cyclic voltammograms of modified electrodes. **S3**

Controlled potential electrolyses. **S8**

Control CPE experiment results. **S11**

Cyclic voltammograms of modified electrodes

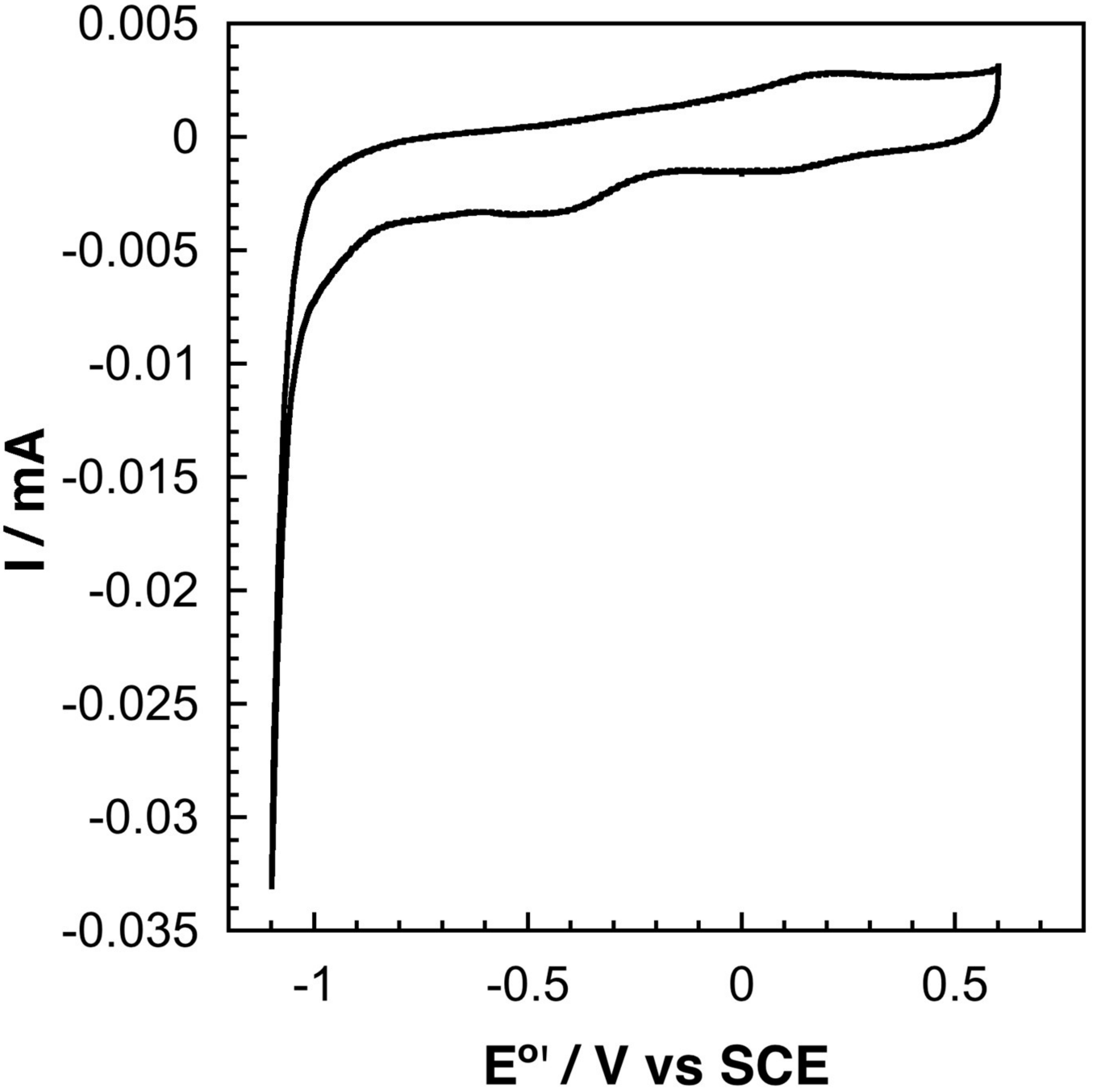

Figure S1. Cyclic voltammogram of CoPc modified edge plane graphite (EPG) electrode at 200 mV s^{-1}.

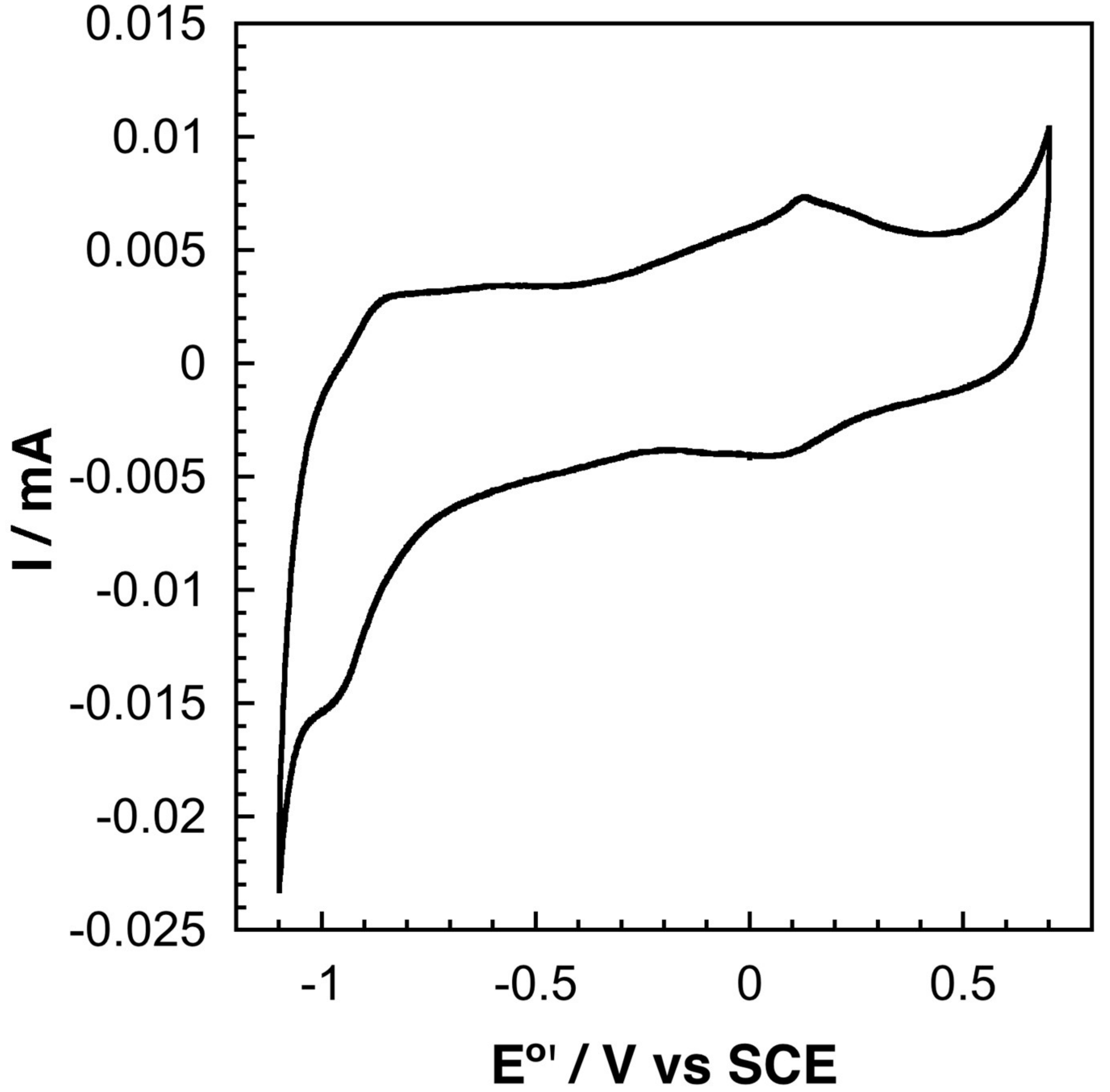

Figure S2. Cyclic voltammogram of CoPc-P4VP modified edge plane graphite (EPG) electrode at 200 mV s^{-1}.

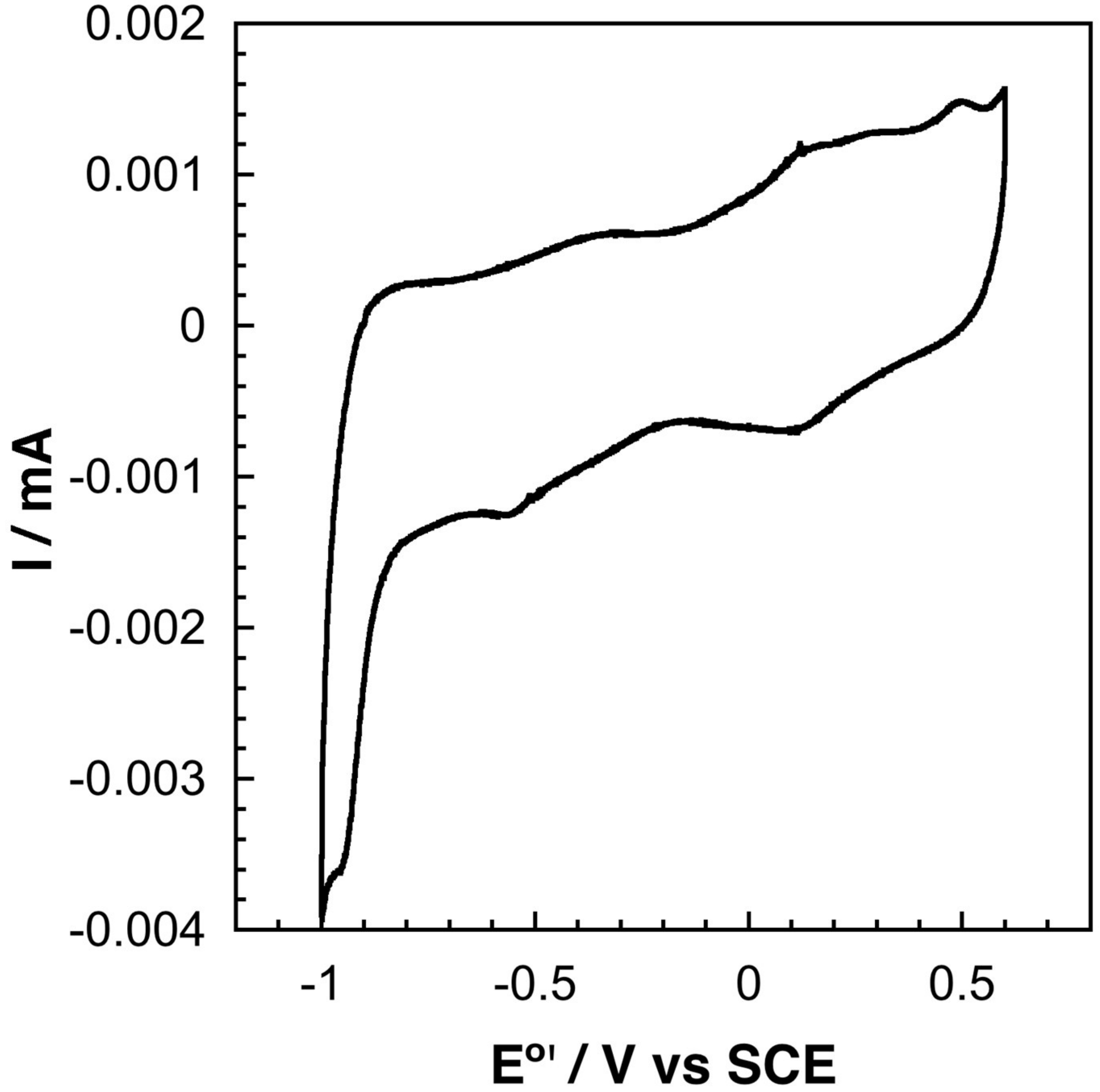

Figure S3. Cyclic voltammogram of CoPc(py) modified edge plane graphite (EPG) electrode at 200 mV s^{-1}.

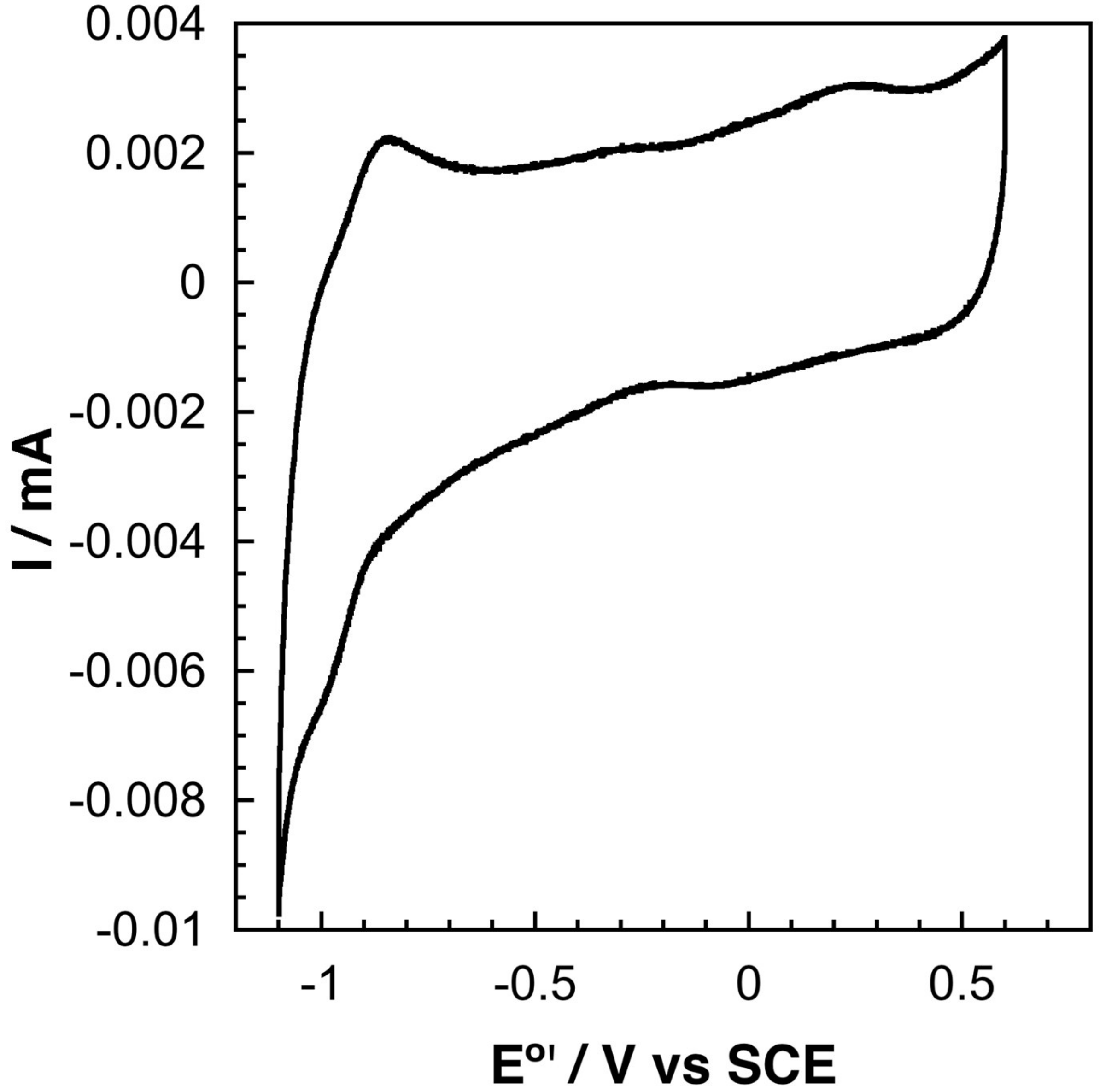

Figure S4. Cyclic voltammogram of CoPc-P2VP modified edge plane graphite (EPG) electrode at 200 mV s^{-1}.

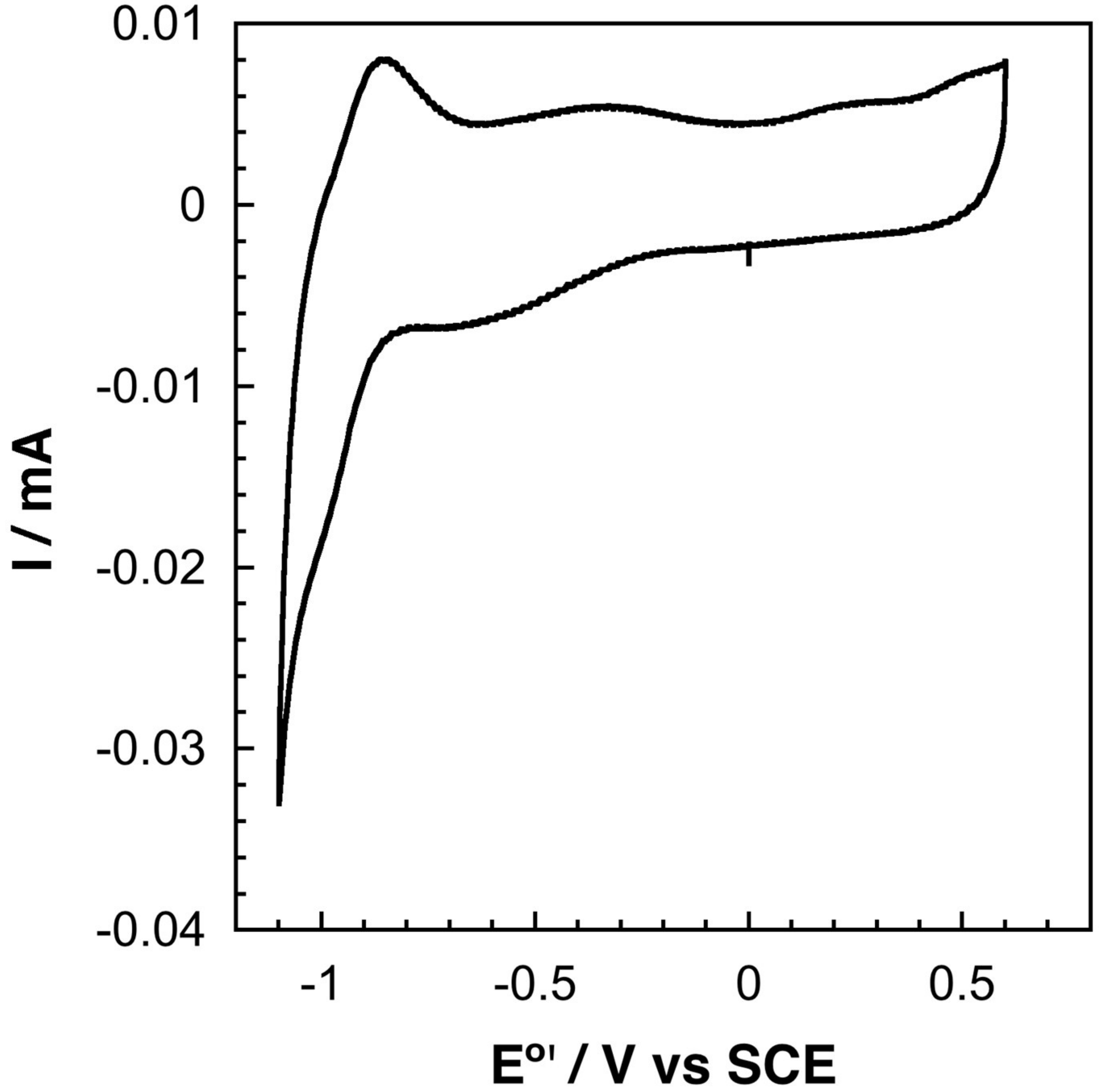

Figure S5. Cyclic voltammogram of CoPc(py)-P2VP modified edge plane graphite (EPG) electrode at 200 mV s^{-1}.

Controlled Potential Electrolyses

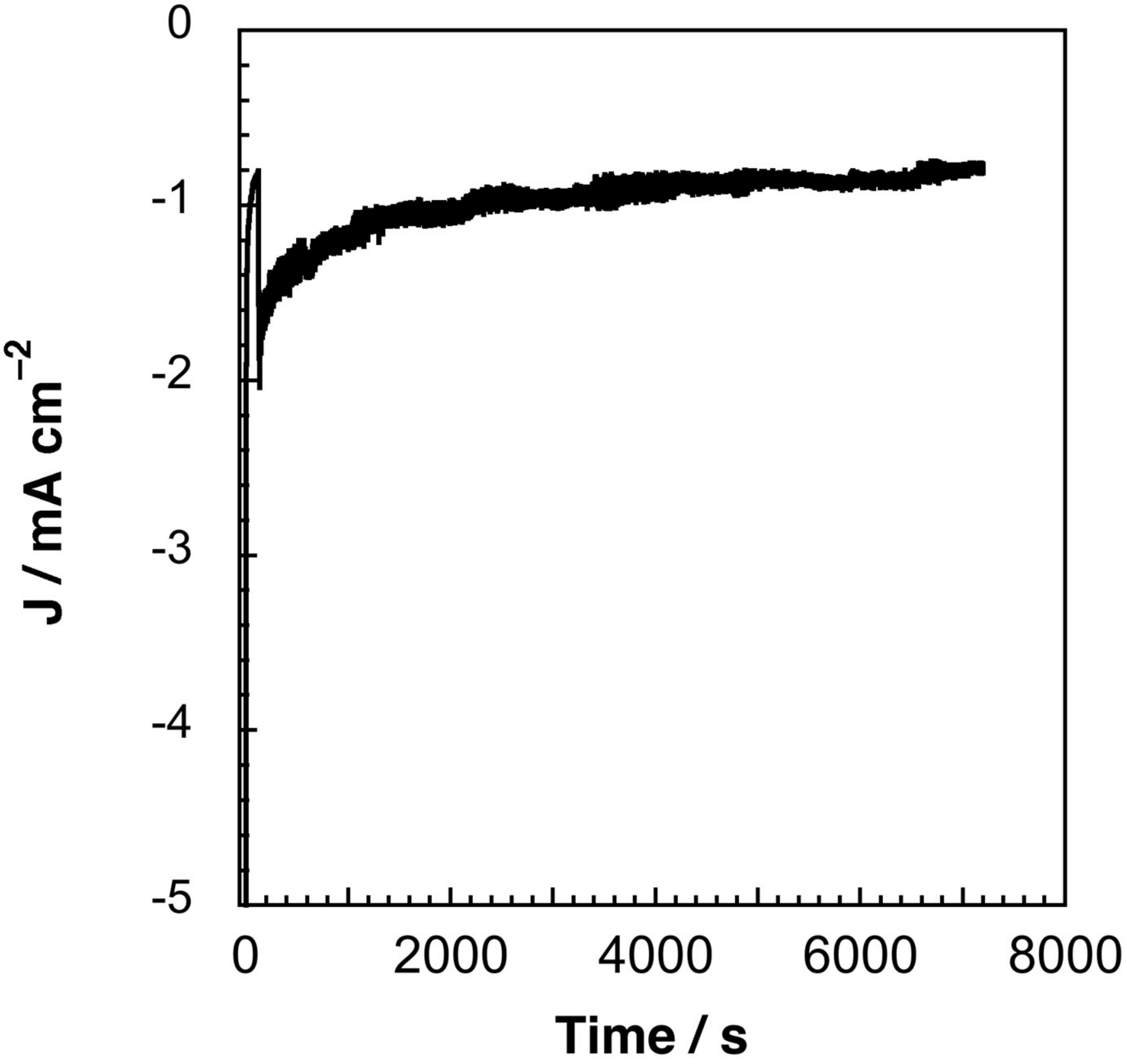

Figure S6. Representative CPE data for CoPc(py) modified edge plane graphite (EPG) electrodes.

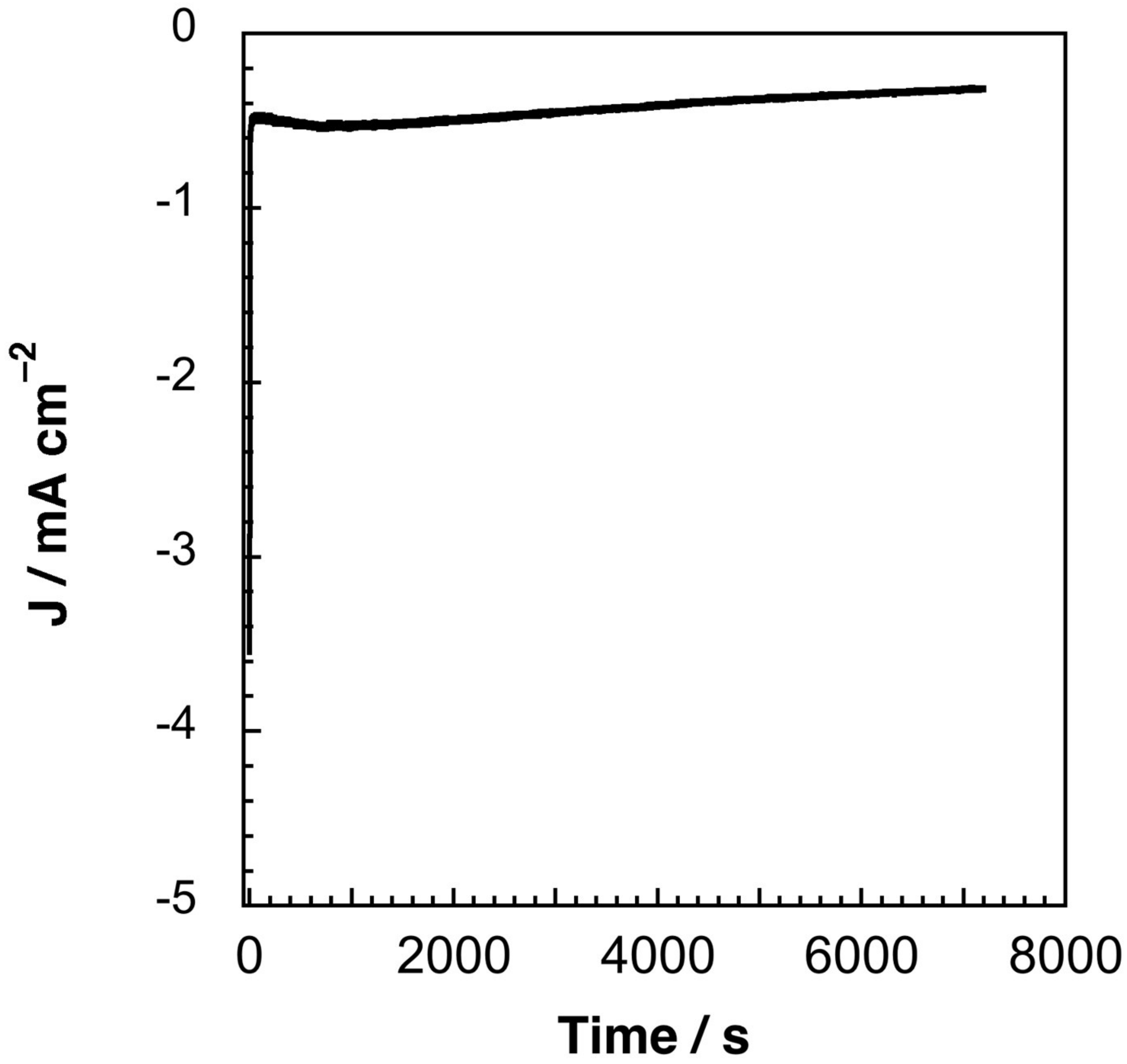

Figure S7. Representative CPE data for CoPc-P2VP modified edge plane graphite (EPG) electrodes.

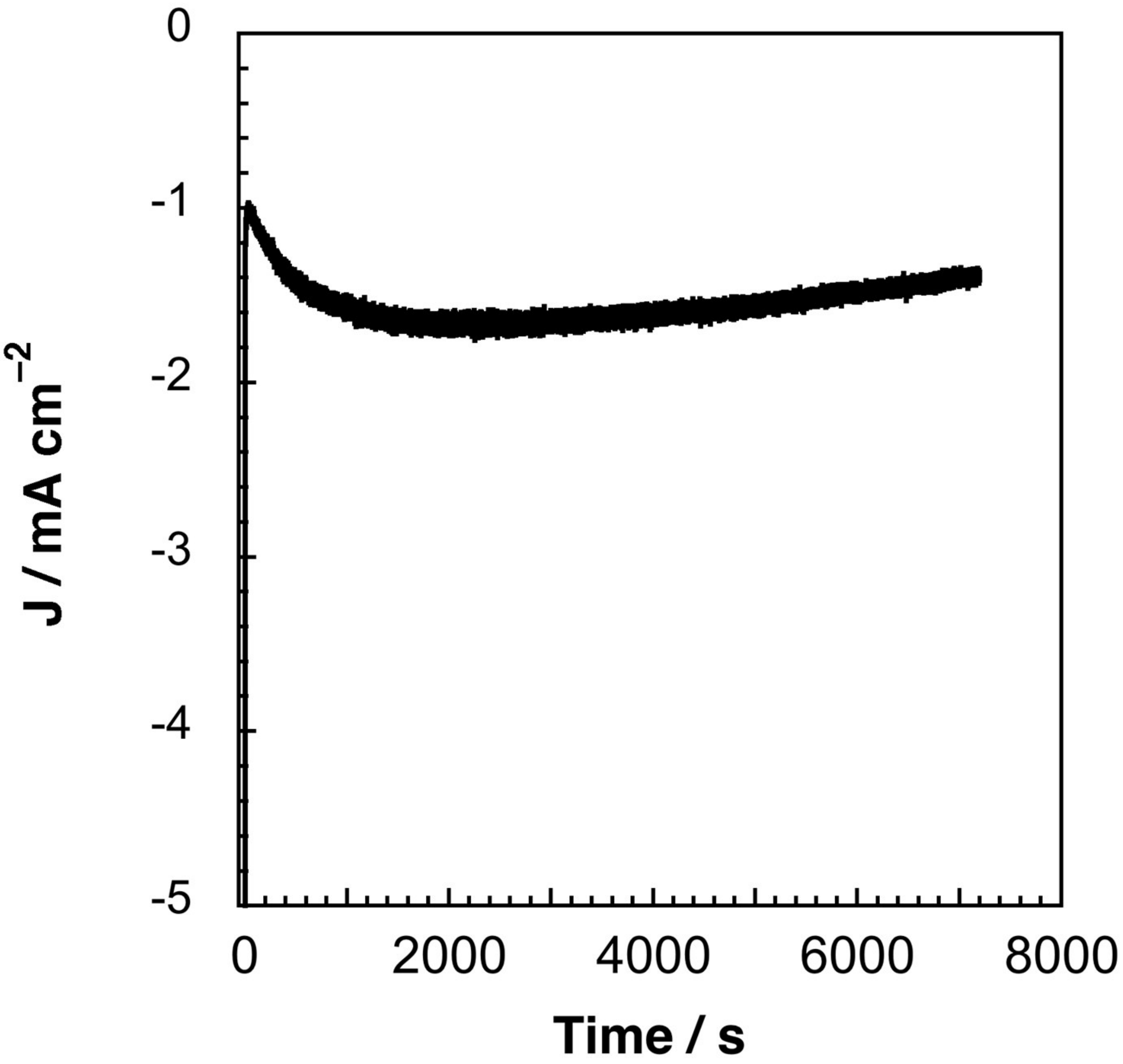

Figure S8. Representative CPE data for CoPc(py) modified edge plane graphite (EPG) electrodes.

Control CPE experiments results

Table S1. Results obtained from 2 h CPE experiments at -1.25 V vs SCE for EPG and EPG-P4VP electrodes in CO_2 saturated 0.1 M aqueous NaH_2PO_4 at pH 4.7 under a CO_2 atmosphere. Results for CPE experiments with CoPc and CoPc-P4VP under the same conditions are included for comparison. Errors are given as standard deviations.

	Charge / C	μmol CO produced	μmol H2 produced
EPG	0.36 ± .0.18	0.17 ± 0.02	1.2 ± 0.8
EPG-PVP	0.061 ± 0.049	0.17 ± 0.2	0.09 ± 0.09
CoPc	0.58 ± 0.24	1.1 ± 0.6	1.2 ± 0.4
CoPc-P4VP	1.9 ± 0.2	8.9 ± 1.1	0.47 ± 0.09

Table S2. Results obtained from 2 h CPE experiments at -1.25 V vs SCE for CoPc and CoPc-P4VP modified electrodes in N_2 saturated 0.1 M aqueous NaH_2PO_4 at pH 5 under a N_2 atmosphere. Results for CPE experiments with CoPc and CoPc-P4VP preformed in CO_2 saturated electrolyte under a CO_2 atmosphere are included for comparison. Errors are given as standard deviations.

	Charge / C	ε_{CO}	TON_{CO} (2h)	ε_{H2}	TON_{H2} (2h)
CoPc (N_2)	0.55 ± 0.29	N/A	N/A	54 ± 16%	$7.4 \pm 5.5 \times 10^3$
CoPc-P4VP (N_2)	0.28 ± 0.16	N/A	N/A	52 ± 16%	$2.4 \pm 1.5 \times 10^3$
CoPc (CO_2)	0.58 ± .24	36 ± 7%	$4.5 \pm 2.4 \times 10^3$	41 ± 8%	$4.7 \pm 1.5 \times 10^3$
CoPc-P4VP (CO_2)	1.9 ± 0.2	89 ± 3%	$3.4 \pm 0.4 \times 10^4$	5 ± 1	$1.9 \pm 0.4 \times 10^3$

Table S3. Results obtained from 2 h CPE experiments at -1.25 V vs SCE for CoPc modified electrodes in CO_2 saturated 0.1 M aqueous NaH_2PO_4 at pH 5 under a CO_2 atmosphere with 0.05 mM added pyridine (py) and 2,6 lutidine (lut). Results for CPE experiments with CoPc without added py or lut and CoPc(py) are included for comparison. Errors are given as standard deviations.

	Charge / C	ε_{CO}	TON_{CO} (2h)	ε_{H2}	TON_{H2} (2h)
CoPc + 0.05 mM py	0.54 ± 0.23	44 ±13%	$5.1 \pm 3.5 \times 10^3$	47 ± 6%	$5.1 \pm 2.3 \times 10^3$
CoPc + 0.05 mM lut	0.55 ± 0.06	32 ± 2%	$3.8 \pm 0.6 \times 10^3$	31 ± 19 %	$3.6 \pm 2.3 \times 10^3$
CoPc	0.58 ± .24	36 ± 7%	$4.5 \pm 2.4 \times 10^3$	41 ± 8%	$4.7 \pm 1.5 \times 10^3$
CoPc(py)	0.83 ± .48	68 ± 3%	$1.2 \pm 0.7 \times 10^4$	19 ± 5%	$2.9 \pm 1.2 \times 10^3$
